# Medical Cannabis in Pediatric Oncology: Friend or Foe?

**DOI:** 10.3390/ph15030359

**Published:** 2022-03-16

**Authors:** Megan Malach, Igor Kovalchuk, Olga Kovalchuk

**Affiliations:** Department of Biological Sciences, University of Lethbridge, Lethbridge, AB T1K3M4, Canada; megan.malach@uleth.ca

**Keywords:** pediatric cancer, cannabinoids, *Cannabis sativa*, medical cannabis, THC, CBD, Epidiolex, Marinol, Sativex

## Abstract

The antineoplastic effects of cannabis have been known since 1975. Since the identification of the components of the endogenous cannabinoid system (ECS) in the 1990s, research into the potential of cannabinoids as medicine has exploded, including in anti-cancer research. However, nearly all of this research has been on adults. Physicians and governing bodies remain cautious in recommending the use of cannabis in children, since the ECS develops early in life and data about cannabis exposure in utero show negative outcomes. However, there exist many published cases of use of cannabis in children to treat pediatric epilepsy and chemotherapy-induced nausea and vomiting (CINV) that show both the safety and efficacy of cannabis in pediatric populations. Additionally, promising preclinical evidence showing that cannabis has anti-cancer effects on pediatric cancer warrants further investigation of cannabis’ use in pediatric cancer patients, as well as other populations of pediatric patients. This review aims to examine the evidence regarding the potential clinical utility of cannabis as an anti-cancer treatment in children by summarizing what is currently known about uses of medical cannabis in children, particularly regarding its anti-cancer potential.

## 1. Background

### 1.1. Pediatric Cancer

Although rare, childhood cancer is one of the leading causes of death among children in Canada and the US. Leukemia is the most common, followed by tumors of the brain and central nervous system (CNS), although the latter is responsible for more pediatric cancer deaths [1,2,3].

The most noticeable difference between adult and childhood cancer is how frequently different types occur. Common adult cancers include those of the lung, colon, pancreas, and breast, while pediatric cancers are overwhelmingly leukemia or CNS tumors [4,5]. Pediatric cancer also differs in its frequency of occurrence, and low participant numbers mean that some types of studies done for adult cancers are not possible with pediatric cancer populations. For this reason, well-designed observational studies are often more practical than clinical trials for studying pediatric cancer, particularly with regard to cancers that are exceptionally rare [6].

The causes of pediatric cancer are less well understood, as the cumulative environmental exposures present in adults are often not present in children. It is thought that pediatric cancers are more often the result of significant/catastrophic errors or abnormalities that occur during normal development. As a result, tumors from an adult and a child with identical histology often have very different biology and, consequently, must be treated differently [6,7,8]. Differences in developing tissues seen in children compared to the established tissues seen in adults may also contribute to a difference in tumor biology [8]. A 2015 pan-cancer analysis of nearly 1000 pediatric tumors found that the somatic mutational burden is much lower in pediatric cancer than in adult cancer, and that significantly mutated genes (genes with driver mutations) were mutated mutually exclusively, which differs from the high co-mutation rate seen in adult cancer. Additionally, only about 30% of significantly mutated genes in pediatric cancers overlapped with previously identified significantly mutated genes for adult cancers [9]. Children also have fewer somatic mutations leading to tumor development, and a subset of pediatric tumors have a high frequency of mutation of epigenetic regulatory proteins [10]. Surprisingly, about 92% of children with cancer do not have pathogenic germline mutations, and 60% have no family history of cancer at all [11].

Importantly, children also respond slightly differently to cancer treatments, such as chemotherapy [12]. Given this, it is not unthinkable that other targeted or novel therapies, including cannabis, may act differently in children compared to adults, and may even act differently between different tumor types in children. Therefore, this warrants the separate investigation of any potential cancer treatments in children as their own distinct group.

### 1.2. Standard Treatment of Pediatric Cancer

As pediatric cancers differ greatly from adult ones, it is not unthinkable that any anti-cancer therapy, including cannabis, may act differently in children compared to adults. The most obvious difference between adult and childhood cancer is how frequently different types occur. Common adult cancers include those of the lung, colon, pancreas, and breast, while pediatric cancers are overwhelmingly leukemia or CNS tumors [4,5]. The causes of pediatric cancer are also less well understood, as the cumulative environmental exposures present in adults are often not present in children. It is thought that pediatric cancers are more often the result of significant/catastrophic errors or abnormalities that occur during normal development. As a result, tumors from an adult and a child with identical histology often have very different biology, and consequently must be treated differently, and indeed may respond differently to the same treatment [6,7,8]. Differences in developing tissues seen in children compared to the established tissues seen in adults may also contribute to a difference in tumor biology [8]. The mutational background of pediatric tumors is different from that of adult tumors. Their somatic mutational burden and the rate of co-mutations is much lower than in adult cancer. Additionally, only about 30% of significantly mutated genes in pediatric cancers overlapped with previously identified significantly mutated genes for adult cancers [9]. Children also have fewer somatic mutations leading to tumor development, and some pediatric tumors have a high frequency of mutation of epigenetic regulatory proteins [10]. Surprisingly, about 92% of children with cancer do not have pathogenic germline mutations, and 60% have no family history of cancer at all [11].

The introduction of chemotherapy to pediatric cancer some 60 years ago has dramatically improved survival rates. However, resistance to chemotherapy is a primary reason for why cancer treatment is unsuccessful in pediatric patients. Additionally, the pharmacokinetics of most anti-cancer drugs have been well studied in adult cancer patients, but the same cannot be said for pediatric cancer patients [13].

Pediatric tumors may also be treated with doses of chemotherapy that exceed “normal” chemotherapeutic treatment. As this treatment is potentially toxic, some of the patients’ immune cells are extracted, frozen, and then returned to the patient after their course of high-dose chemotherapy is finished. Typically, relapsed or refractory patients receive this treatment [14]. This high-dose treatment may allow some chemotherapeutic agents to better penetrate the blood–brain barrier, improve the dose–response effect [15], and allow young patients to avoid the damaging effects of radiotherapy [14,16], though radiotherapy may also be combined with HDC-SCR to “salvage” relapsed patients [15]. The neuropsychological effects of HDC-SCR have been found to be minimal, and younger patients are less susceptible to these effects [17]. Patients with brain tumors who undergo this treatment may also suffer few post-transplant infections [16]. While the treatment is intense, the average survival rate of patients improves from 40–50% to 60–69% with this treatment [14]. 

Unfortunately, the intense nature of HDC-SCR regimens means that nearly all patients who undergo them suffer from severe side effects, particularly high-grade blood toxicities. It is also likely that available HDC-SCR regimens have likely reached their maximum possible intensification, as further dose increases will likely only increase toxicity with no change in clinical usefulness [14]. 

Exposure to ionizing radiation is one of the few recognized environmental causes of childhood cancer, and for this reason, it is avoided as much as possible in pediatric cancer treatment [3,18]. As a result, the use of radiation in the treatment of pediatric cancer patients has decreased drastically over the past 30 years. Where radiation must still be used during treatment, improvements in medical radiation technology that allow for more targeted applications have made it safer for pediatric patients. Improved diagnosis and prognosis of pediatric tumors also ensures that radiation is mainly used in cases where the tumor severity warrants aggressive treatment, and not in cases where the tumor may be successfully treated with less aggressive options [19,20].

It is estimated that as of 2020, there were more than 500,000 living survivors of childhood cancer in the US [21]. Even after surviving cancer and the toxicities associated with its treatment, which they typically tolerate better than adults, these survivors often suffer late effects: sequelae resulting from treatment that are not realized until years or decades later. These sequelae include auditory, oral, and dental complications, neurocognitive impairment, including deficits in learning and memory, cognition, and high executive functions [22], impaired function of cardiovascular, pulmonary, gastrointestinal, and genitourinary systems, thyroid, growth, and musculoskeletal issues, and issues with gonadal hormones that may affect sex development and fertility. This treatment may also result in the development of subsequent cancers; the exact nature of the subsequent tumor depends on the type and dose of chemotherapeutic agents and the location and dose of radiotherapy, as well as the age of the patient during treatment [3]. 

While historical studies show the risks associated with cancer treatment of previous eras, it is not yet known how survivors treated with modern treatments will fare, partially because there are not yet any clear guidelines for how to follow up with survivors to evaluate late effects [21]. While the types of drugs used in cancer treatment remain largely the same as they did up to 50 years ago, significant changes have been made to recommended dosing and scheduling. Regarding radiation, improvements in technology have made it more targeted, and it is used less frequently, as discussed above [19,20]. Together, these suggest that survivors today may be somewhat less likely to develop chronic conditions from their treatment.

Given the doses of many chemotherapeutic agents administered to cancer patients and the toxicities associated with them, most oncologists spend a majority of their time on supportive care; that is, care related specifically to managing the symptoms that occur as a result of cancer treatment [13]. For all of these reasons, new therapies are desperately needed for childhood cancer to improve survival and reduce the toxic side effects of treatment.

### 1.3. The Endocannabinoid System and the Developing Brain

The endogenous cannabinoid system (ECS) and the opioid system in the human body have remained through several million years of human evolution, and it is possible that the ECS evolved before the cannabis plant itself [23]. The two main ECS receptors in humans are CB1 and CB2. Discovered and cloned in the early 1990s, both are G-protein coupled receptors. While CB1 is found mostly in the brain and CNS, CB2 is found mainly in immune cells and tissues [24,25,26]. Their endogenous ligands are the lipids known as N-arachidonoyl ethanolamine (AEA) and 2-AG (2-arachidonoylglycerol) [24]. The therapeutic effects of agonism and antagonism of both receptors have been found to be clinically useful for a plethora of disorders [24,27]. However, the endocannabinoid system involves many more receptors and ligands and has therefore been termed the “endocannabinoidome.” The scale of the endocannabinoidome makes it a promising therapeutic avenue for many disorders, including cancer [24]. Other receptors that have been discovered to be part of the endocannabinoidome include transient receptor potential cation channel vanilloid receptors (TRPV) 1 and 2 [28], peroxisome proliferator-activated receptor gamma (PPARγ), and previously orphaned G-protein coupled receptors GPR18, GPR55, and GPR19. The endogenous cannabinoids AEA and 2-AG have been demonstrated to be active on some of these receptors as well [29].

In addition to the effects of endogenous cannabinoids in the body, *Cannabis sativa* plants produce phytocannabinoids that act on the above receptors in the body in different ways [29]. These include the well-known tetrahydrocannabinol (Δ^9^-THC) [30] and cannabidiol (CBD) [31], first identified nearly 60 years ago. These two phytocannabinoids remain the most well studied. However, cannabis also contains many other phytocannabinoids, such as cannabidivarin (CBDV) [32], cannabinol (CBN), cannabigerol (CBG), cannabivarin (CBV), cannbichromene (CBC), and tetrahydrocannabivarin, along with numerous other terpenoids and flavonoids [33].

### 1.4. The Endocannabinoid System and Early Brain Development

There is concern that any cannabis-based treatment received by children will impact their brain development, as the major components of the endocannabinoid system in the brain, including functional CB1 receptors and the endocannabinoids 2-AG and AEA, are present around gestational week 19 in humans and likely have a role in critical early behavior processes [34,35,36]. Evidence from rats also indicates that more than simply being present, CB1 receptors in the developing fetal brain are functional [37]. This system may also contribute to the proper formation, growth, migration, and wiring of various areas in the developing fetal brain [34,38,39]. The expression of the CB1 receptor in the brain also changes throughout human development. Both the expression levels and locations of expression differ greatly between fetal brain development and early life when compared to adult brains [35,36]. Any evaluation of cannabis-based treatments in children, then, needs to evaluate the potential effects of this treatment on the brain development of children, as they have a functional ECS that has the potential to be disturbed and altered using cannabis-based treatments. There are numerous existing protocols and policies for following the late effects of childhood cancer on survivors in order to better modify existing treatment plans for future patients to minimize toxicity and treatment-related morbidity and mortality [18]. It is not unthinkable that cannabis-based therapies and drugs could fit nicely into these existing structures.

### 1.5. Anti-Cancer Mechanisms of Phytocannabinoids

Δ^9^-THC was first found to have anti-cancer effects in Lewis lung adenocarcinoma in 1975 [40]. Interest in the potential of cannabis for cancer treatment has resulted in significant research in the last decade [41]. The most prevalent cannabinoids, Δ^9^-THC, CBD, and CBN, have been shown to affect growth and proliferation of cancer cells [24,29]. Most research on the effectiveness of cannabis for use as a curative agent in cancer focuses only on the two main components, THC and CBD, or their synthetic analogues; very little has been published on the pharmacology or potential clinical use of terpenoids and flavonoids, which are also major components in cannabis [23,29]. Whole plant extracts are known to have other active molecules that may also have cytotoxic properties, and consequently, full extracts exhibit so-called combined “entourage effects” [29,42]. Additionally, different combinations of phytocannabinoids can be more effective than the use of single cannabinoids [43]. It is also possible to grow cannabis strains with a particular composition to best suit the needs of a patient, whether in terms of their age, symptoms, or tumor type. The fact that different combinations of phytocannabinoids can be more effective is believed to be due to the “entourage effect” that many other components of cannabis, such as terpenoids and flavonoids, may have on the actions of CBD and THC [23,44]. In fact, recent research has shown that whole cannabis extract is more effective than pure THC alone at reducing the growth of cancer cells of many different types of cancer [42]. Despite this evidence that a more heterogeneous mix of cannabinoids may be more effective than pure cannabinoids alone, no cannabis lines have yet been developed and tested specifically for cancer treatments.

Numerous recent reviews have detailed the preclinical evidence in support of the anti-tumor action of cannabinoids in a variety of adult tumors. While it is beyond the scope of this review to fully evaluate this evidence, the following reviews provide an excellent summary [41,45,46,47,48,49,50,51].

**Figure 1:**
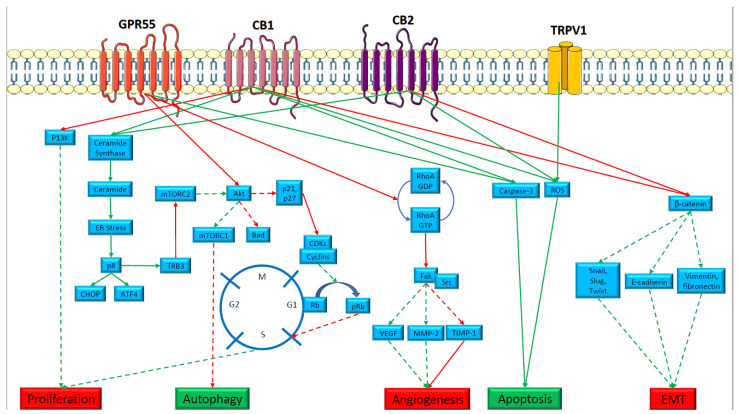
The mechanisms by which cannabinoids have anti-cancer effects.

The mechanisms by which cannabinoids exert anti-cancer effects have been thoroughly investigated and include the inhibition of angiogenesis, the induction of apoptosis and autophagy, and the slowing of rapid cell growth (Figure 1). Cannabinoids seem to use a variety of well-known and well-understood pathways associated with these phenomena, including the induction of apoptosis via an increase in reactive oxygen species (ROS), the MAPK pathway, increased ceramide production and the ER stress pathway via p8, activation of caspase 3, and inhibition of protein kinase B (AKT), the induction of autophagy via the ER stress and AKT pathways, the inhibition of excessive proliferation via the phosphoinositide-3 kinase pathway and through decreases in cyclic adenosine monophosphate (cAMP) via inhibition of adenylate cyclase, inhibition of the epithelial–mesenchymal transition (EMT) via inhibition of β-catenin, and inhibition of angiogenesis via inhibition of Ras homolog family member A (RhoA) GTPase leading to inhibition of downstream proteins such as focal adhesion kinase (FAK), vascular endothelial growth factor (VEGF), and matrix metalloproteinase-2 (MMP-2) (Figure 1). These effects seem to be directly mediated through CB1 and CB2 receptors, as well as other GPCRs potentially included in the ECS, as mentioned above (Figure 1) [24,29,41,45,47,48,51]. This anti-cancer response seems to vary by tumor type, stage, and even the molecular background of the tumor [29].

Conversely, a small number of studies have suggested that the endocannabinoidome may contribute to the development, progression, or prognosis of cancer. High levels of CB1 and AEA in tumors have been found to overexpress genes that promote cancer growth [52]. The CB1 receptor may also activate the chimeric oncogenic transcription factor PAX3-FOXO1, increasing cancer cell growth [53]. Treatment of cancer cells with a variety of synthetic and plant-derived cannabinoids was found to increase cell proliferation by upregulating the ERK1/2 and Akt survival pathways via EGFR activation [54]. High CB1 and CB2 levels have also been found in cases of chronic lymphocytic leukemia (CLL) [55]. Many TRP channels are upregulated in different cancers, and the potential to exploit them in cancer treatment, though not with cannabinoids, has been explored [56].

Most of these molecular pathways and anti-cancer effects, while promising, have only been shown to function in adult cancer. Studies confirming these or other molecular anti-cancer effects of cannabinoids in pediatric cancer cells are scarce, and given the differences between pediatric and adult tumors and the role of the ECS in brain development in children, the anti-cancer effects of cannabis need to be investigated in pediatric cancer specifically.

## 2. Potential Uses of Cannabis in Pediatric Oncology

When considering cannabis-based drugs or medical marijuana, CBD is a popular choice for use in pediatric populations because it does not have the psychoactive side effects of THC [57]. Most of the research involving the use of cannabis-based drugs or medical marijuana in children has been on treatment for epilepsy, followed by chemotherapy-induced nausea and vomiting or otherwise controlling the unpleasant side effects of cancer and its treatments [58,59].

Numerous clinical trials in past decades have investigated cannabis-based drugs, but especially those with CBD, for the control of pediatric epilepsy (Table 1 and Table 2), though the mechanism by which CBD reduces seizures remains unknown [57]. 

Clinical studies of CBD-based drugs on pediatric epilepsy demonstrate that CBD is safe and effective for controlling seizures in children with a variety of epileptic disorders, whether alone or concomitantly with other antiepileptic drugs (AEDs) [63,64,65,66,67,68,69,71,72,73]. Non-serious adverse events can largely be resolved by reducing the dose of CBD, the concomitant AED, or both [74,75,76,77]. Studies of THC-based drugs have similar findings and did not find evidence of habituation, a frequent concern when prescribing cannabis-based drugs to this patient population [61,78].

The use of cannabis for these disorders indicates that cannabis can be used in children in a safe and effective way with minimal side effects and low risk of serious adverse events. This provides a strong rationale for investigations of cannabis’ potential effectiveness in treating other conditions in pediatric patients, particularly where the evidence of cannabis’ effectiveness is strong in adults with the same condition. It also assuages concerns about the safety of using cannabis in a pediatric population, a factor which often stands in the way of physicians recommending cannabis, medical marijuana, or cannabis-based drugs to this patient population. Regarding pediatric cancer, the anti-cancer effects of cannabis, combined with minimal side effects and control of CINV, make it an attractive treatment option for pediatric cancer.

While there are legitimate concerns about the use of medical marijuana in pediatric populations based on evidence from studies using mice or healthy human participants, the decision of how best to treat a severely ill child, especially one who is terminally ill, alters the risk/benefit analysis done before deciding upon a therapy [59]. At present, there is no conclusive literature regarding the use of cannabis in pediatric cancer care [79,80].

Most studies where pediatric patients are given cannabis-based drugs involve oral administration, typically oil drops or oromucosal sprays (Table 1 and Table 2). While obviously preferable to inhalation, given the age of the patients, this method typically has low bioavailability, typically between 5% and 20% [81]. While intravenously administered cannabis has similar bioavailability to inhaled cannabis [81], there have been no investigations into this method of administration in children.

Cannabis has only been anecdotally reported to kill or treat cancer in children. All other data are currently preclinical from either cell lines alone or both cell lines and animal xenograft models [79,80]. However, given the differences between pediatric and adult cancer discussed earlier and the fact that the ways in which cannabinoid agonists react with receptors may differ between species and between in vitro and in vivo experiments [82], it is important to study cannabis specifically in a pediatric clinical trial setting to get a good sense of its effectiveness and potential side effects that may be specific to pediatric use [80].

### 2.1. Currently Available Cannabis-Based Drugs

The US currently has only one approved CBD medication for pediatric patients, Epidiolex. This drug has been well studied, including in clinical trials, and is considered safe to use in the treatment of young patients with refractory seizures [83]. This drug has been the subject of many clinical trials for many different epileptic disorders in children (Table 1), most of which have found that it reduced seizure frequency with few side effects [74]. Most investigation into the use of medical marijuana in children is done in the context of epileptic disorders, and currently available evidence examining the use of cannabis in pediatric oncology patients is limited to case studies and descriptive studies, rather than clinical studies or trials.

### 2.2. Use of Cannabis in Chemotherapy-Induced Nausea and Vomiting

Cannabis is frequently suggested as an alternative therapy that may improve already poor symptom control for oncology patients, particularly for control of chemotherapy-induced nausea and vomiting (CINV) [23]. The investigation of cannabis as an antiemetic in children goes back to the 1980s; however, there have been relatively few studies on this phenomenon since then. Cannabis has been found to be an effective antiemetic in children when compared to placebo or conventional antiemetics, with side effects similar to those reported in adults. Most of these cannabis products were high-THC ones [58]. However, this effect has not been conclusively replicated in multiple studies [59].

A 2016 review of studies of antiemetic agents in children found only 34 suitable studies and found 5-HT3 agonists combined with dexamethasone to be the most effective treatment, although they insist that there is generally a dearth of research into this area. Regarding cannabinoids, they state they are probably effective, but have many side effects, mostly negative, though some other more positive side effects were also reported by patients. This study found only three clinical trials comparing cannabis-based antiemetic drugs to standard antiemetic drugs [84]. These studies are reviewed below.

Two of the three clinical trials found nabilone, a synthetic THC analog, more effective than two standard anti-nausea agents, domperidone [85] and prochlorperazine [86]. In both studies, nabilone was significantly more effective at reducing episodes of emesis and was preferred by significantly more patients and/or parents. However, in both cases, more side effects were reported with nabilone. In one study, a reduction in the dosage of nabilone completely eliminated all major side effects, and patients noted a faster return to normal and shorter hospital stay after treatment with nabilone [86]. It is notable that nabilone was preferred in both studies, despite serious side effects that exceeded those of already standard antiemetic medications.

In the third trial, Δ^9^-THC was tested against metoclopramide and perchlorazine and was found to be a significantly better anti-nausea and anti-vomiting medication than both [87]. It was also found to decrease anorexia and increase appetite. As with nabilone, the most common side effect was drowsiness, although the increase in this effect was only significant between THC and metoclopramide. Only two patients reported feeling any kind of “high” on this treatment, and this resulted in one patient stopping treatment. 

Δ^8^-THC, an isomer of THC that differs from Δ^9^-THC, was also tested on eight children with different hematological cancers on different treatment regimens expected to produce vomiting. The authors found that THC completely prevented all vomiting across a variety of chemotherapy cycles among all eight patients; they also observed a complete lack of side effects [88].

Taken together, all these results indicate the use of medical cannabis or synthetic cannabinoid drugs as antiemetics and anti-nausea medications is worth exploring with pediatric patients under careful supervision, especially if traditional antiemetic and anti-nausea medications are ineffective for a patient.

### 2.3. Effect of Cannabis-Based Drugs on Quality of Life

A common use of medical marijuana in pediatric populations, particularly in cancer populations, is palliation to increase patient comfort and quality of life during difficult and intense treatments. A descriptive study of the use of medical marijuana in a hospital in Israel for pediatric cancer patients showed a high (80%) satisfaction rate among patients and parents for alleviation of symptoms related to both their disease and treatment, with no negative side effects reported. However, some patients did not respond to medical marijuana treatment. The authors attributed the lack of negative side effects (and possibly the lack of response) to the fact that most chose oil drops as the method of ingestion, which typically have very low bioavailability [81]. However, the study did not use validated methods of collecting information about patient response and acknowledges the possibility of a placebo effect [89].

At Children’s Hospital Colorado, there is currently a prospective observational study recruiting pediatric CNS tumor patients to examine their quality of life while using marijuana-derived products concurrently with either curative or palliative treatment (NCT03052738).

### 2.4. Cannabis’ Anti-Cancer Potential: Pediatric Brain Tumors

Research using pediatric brain tumor cell lines to investigate both the molecular anti-cancer mechanisms and the potential of cannabis as a treatment is rare but does exist and is done almost exclusively in neuroblastoma models (Table 3). A retrospective molecular analysis of pediatric low-grade gliomas at one institute found that tumors that spontaneously involuted or remained stable had higher levels of the mRNA coding for CB1 (CNR1) than tumors that regressed. The authors hypothesized that the potential anti-cancer pathways triggered by the activation of CB1 receptors may represent a mechanism for this effect, while also acknowledging that the types of tumors in these cases may spontaneously go into remission [90]. Similarly, CB2 receptors have been found in pediatric brain tumors of many types, both malignant and benign, except for embryonal tumors. The level of expression CB2 receptors correlated with tumor malignancy, and the highest levels of receptors were found in areas containing high levels of very proliferative and invasive cells [91]. While troubling, this study does indicate that CB2-mediated anti-cancer mechanisms are also a possible avenue of treatment in these tumors.

Regarding the mechanism by which cannabis may act on pediatric brain tumors, there are four studies. A 2016 study found that CBD was able to decrease the viability and induce apoptosis of neuroblastoma cells, in addition to decreasing tumor growth with in vivo xenografts [92]. As CBD does not have a particularly high affinity for CB1 [27], this further suggests that anti-cancer effects may be triggered by making use of receptors other than CB1. CBD was also found to affect miRNA expression in neuroblastoma, downregulating hsa-let-7a and upregulating hsa-miRNA-1972. These altered miRNAs resulted in altered levels of protein, with hsa-let-7a increasing levels of apoptosis proteins caspase-3 and GAS-7 and with hsa-miRNA-1972 decreasing anti-apoptotic proteins BCL2L1, SIRT1, and MYCN. CBD in this cell line also decreased levels of AKT1, increased PTEN, caused apoptosis, inhibited migration, and shifted cellular metabolism towards glycolysis [93]. One synthetic cannabinoid, AM404, has been investigated in neuroblastoma as well. Though not demonstrated to inhibit cell viability, it inhibits invasion via decreased expression of MMP-1, 3, and 7 and inhibits transcriptional activity of pro-tumor transcription factors NFAT and NF-κB through CB1 and TRPV1-independent pathways [94].

Regarding clinical indications of the effectiveness of cannabis, a descriptive study of pediatric cancer patients who chose to use medical marijuana at a children’s hospital in Israel found no indications that anti-convulsants and medical marijuana interacted negatively in children with brain tumors [89]. THC inhalation by smoking recreationally was suspected to be involved in the regression of two pediatric pilocytic astrocytomas, as the patients received no treatment outside of initial subtotal surgical resection. However, the tumors had low proliferative indices, and regression for low-grade pilocytic astrocytomas is a known occurrence, making this evidence relatively unconvincing [102]. These studies establish that cannabis can be non-toxic in children with cancer in appropriate doses and that cannabis does have the clinical potential to be a cancer treatment, pending more detailed investigation.

### 2.5. Cannabis’ Anti-Cancer Potential: Pediatric Leukemia

Much of the investigation into the effects of cannabis in pediatric cancer preclinically have been in leukemia (Table 3).

THC has received the most attention as an anti-cancer agent in preclinical leukemia models. In 2005, researchers found that THC induced apoptosis in Jurkat cells, a human T cell line. This apoptosis was triggered by the extrinsic pathway through cleavage of caspases 8 and 10 and by the intrinsic pathway through the alteration of the mitochondrial membrane potential and release of cytochrome c [96]. Another investigation into the mechanism of THC-triggered apoptosis in Jurkat cells found that it also interferes with the Raf-1/MEK/ERK signaling pathway, mainly by altering the phosphorylation status of these molecules. This triggered the relocation of BAD to the mitochondria, resulting in activation of the intrinsic apoptotic pathway [97]. Another study also found that THC can induce apoptosis of Jurkat cells via ceramide biosynthesis, triggering the same intrinsic pathway via the mitochondria. Jurkat cells, as immune cells, express primarily CB2 receptors, and all of the above effects were found to be mediated via CB2 [98]. This is notable, as CB1 is the receptor through which THC-induced psychoactivity, a major concern when considering the use of cannabis in pediatric populations, is mediated. More recent studies have investigated the use of the THC-analogue CPP55940 in Jurkat cells and found that it induces apoptosis via the production of reactive oxygen species (ROS), which triggers the intrinsic apoptotic pathway via a CB1- and CB2-independent mechanism, and that, notably, this does not occur in normal peripheral blood lymphocytes [99].

CBD has also been shown to induce apoptosis in leukemia cells. It has been demonstrated to act on mitochondria to induce cell death both via apoptosis and autophagy in a variety of T-ALL models [101]. It also decreases viability and triggers apoptosis in vitro and in vivo via an increase in ROS via CBD’s action on the CB2 receptor [95]. There is also evidence in leukemia of an entourage effect wherein cannabinoid combinations consisting of two of CBD, CBG, and THC have been found to work synergistically with the chemotherapeutic agents vincristine and cytarabine, especially for a CBD+THC combination. Particularly interesting is the finding that using cannabinoid pairs after using either of the chemotherapy agents was more effective than using the cannabinoid pair and chemotherapy simultaneously [43].

There has been some evidence of cannabis’ anti-cancer activity in patients. A 2013 case study by Singh and Bali found evidence of anti-tumor activity in the case of a 14-year-old patient with aggressive acute lymphoblastic leukemia (ALL). The patient had a Philadelphia chromosomal translocation, which is a poor prognostic marker in ALL. After intense treatment, the patient was recommended for palliative care. At this point, the family began treatment with numerous different hemp oils and worked to increase the frequency and concentration of doses over time. Many decreases in blast cell counts were closely correlated with increased dosages, meaning that the results cannot be attributed to spontaneous regression. Additionally, many of the negative side effects of chemotherapy were not observed with hemp oil treatment, and the patient reported an improved quality of life while being treated with hemp oil. While the patient eventually died from complications related to their disease, the authors speculate that the aggressive nature of the prior treatment may have been related to the cause of death. The authors also note that prior aggressive chemotherapy had failed to decrease blast cell counts and had devastating side effects, while hemp oil treatment had done the opposite [103]. Another investigation into the expression and potential clinical utility of CB1 and CB2 in chronic lymphocytic leukemia (CLL) patients found that high CB1 expression (as measured via mRNA levels) was correlated with poor outcomes, and cultures of patient cells and normal cell controls from healthy patients showed decreased viability in response to cannabinoid treatment (primarily synthetic). This effect was not correlated with expression levels of CB1 or CB2, suggesting the involvement of other receptors despite the use of synthetic cannabinoids designed to target CB1 or CB2 specifically [55].

### 2.6. Cannabis’ Anti-Cancer Potential: Other Pediatric Tumors

A 2009 study found that CB1 gene expression is significantly upregulated in rhabdomyosarcoma, a rare soft tissue cancer in children, and stimulation of CB1 with THC and HU-210, a synthetic THC analogue, were able to decrease the viability of several rhabdomyosarcoma cell lines. This was mediated via Akt pathway activation triggering increased caspase 3 and therefore apoptosis, as well as increased activation of p8. Caspase 3 activation and decreases in viability were also observed in vivo [101].

## 3. Concerns about Use of Cannabis in a Pediatric Population

### 3.1. Early Cannabis Exposure and Brain Development

Disturbing the ECS during prenatal and early postnatal development has been shown to have drastic behavioral consequences for both rats and humans, with some effects persisting into adulthood and others vanishing as an adult phenotype is reached [39,104]. At present, adolescence seems to be a highly delicate time for cannabis use; the development of appropriate stress responses and connectivity between various regions of the brain can be significantly impacted by its use [105,106]. This developing ECS may also be to the benefit of pediatric patients, as many endocannabinoid system researchers have theorized that because the endocannabinoid system is not yet fully developed in younger patients, cannabis treatments that make use of THC or CB1 agonism may not produce the undesirable side effect of psychoactivity [39,91].

### 3.2. Lack of Clinical Studies

Most studies conducted on medical cannabis and children have issues with sample sizes and adequate controls, meaning that physicians have little to no high-quality information to use when considering prescribing medical cannabis [58,59]. This is a major issue in the preclinical literature as well, where medical marijuana studies are focused primarily on adults.

However, things may be improving. A recent review of publicly available evidence regarding cannabis in pediatric cancer care found that the majority of articles available were of at least satisfactory quality and emphasized a need for further research, particularly case studies that could more exactly determine efficacy, dosage, and exact formulation [79].

### 3.3. Potential for Addiction

Though there is concern regarding the addictive potential of marijuana and its ability to alter brain development with consequences that are still not fully determined, much of the research in this area has been done on adolescents and young adults. While adolescents are considered pediatric patients, their brain development is different from that of a child, and risks of using medical marijuana or cannabinoid-based therapies in this time period are not generalizable to children [59]. One clinical trial has evaluated the risk of habituation with Δ^9^-THC treatment in pediatric patients and found no risk of habituation [78].

Additionally, cannabis treatment in children does have the potential to alter other forms of addiction. Studies in mice and rats indicate that prenatal exposure to Δ^9^-THC may alter the potential for adults to become addicted to opioids by altering the development of opioidergic neurons [35].

### 3.4. Physician Perspectives on Pediatric Cannabis Use

Publicly accessible data on the use of cannabis in pediatric cancer often provide reasons for why to use or not to use it. Reasons against use include developmental delays and addictive potential, potential interactions with other drugs, stigma, and the supposed “absence of benefits.” Reasons for its use are more common, with a focus on alleviating the side effects of chemotherapy, such as nausea, vomiting, pain, and anxiety or other psychosocial concerns. Nearly half of the articles claim that cannabis could kill cancer, despite the fact that research indicating this is largely preclinical and there are no conclusive published pediatric clinical trials using cannabis to date [79].

Another common issue seen in the available literature concerns the advice of physicians. Many physicians have been found to be against family decisions to use cannabis based on a lack of evidence regarding efficacy, dosage, and formulation [79]. Interestingly, other research into US physicians’ opinions on cannabis use in pediatric oncology patients indicates that physicians are very interested in cannabis and are willing to help these patients access medical marijuana despite concerns about how exactly to go about doing so [107]. There is also some suggestion that physicians are against the use of cannabis simply because they are unaware of the potential benefits of cannabis or cannabinoid therapies [23]. Unfortunately, physician unwillingness to prescribe, recommend, or even condone cannabis for patients leads them to seek it from potentially illegal sources, or to try ineffective strains that may have highly undesirable side effects [23].

As of now, there is little to no information on important physician considerations, such as dosing, risks and benefits, medication interactions, and side effects [108]. Formulations and doses of cannabis are likewise completely uncontrolled, as the cannabis industry remains private and unregulated. This means that a physician recommending medical marijuana will not be able to write a specific prescription at a specific dose or even recommend a specific strain [59]. For these reasons, physicians are curious but cautious and likely cannot recommend cannabis to pediatric patients until pediatric-specific trials that examine the effect of this use not only on killing the cancer, but also on side effects, brain development, and other potential long-term sequelae have been conducted [79].

### 3.5. Reports of Adverse Events with Pediatric Cannabis Use

As discussed earlier, CBD is a popular option for use in children due to its lack of psychoactive side effects, current use in palliation, and low toxicity [27,83,108,109]. However, many CBD products may not be as pure as claimed, nor are they always labeled with the exact amount of CBD present, which may lead to patients unintentionally experiencing undesirable side effects from imprecise doses of CBD [83]. Additionally, some evidence that CBD may be able to bind, though not necessarily agonize, the CB1 receptor raises concerns that it may interfere with any CB1-mediated processes [109].

In the US, there were 518 CBD poisonings reported in 2018 and 492 by May 2019 [83]. These may have been the result of inaccurately labeled products or miscalculated dosages and illustrate that CBD is not a harm-free product, which may explain why physicians remain cautious about recommending CBD use.

Nearly all trials concerning the use of cannabis-based treatments for CINV and epilepsy mention some study participants dropping out due to severe side effects of cannabis treatment. A common concern in reviews concerning pediatric patients and cannabis is the increased levels of side effects that occur with cannabis-based treatments, regardless of whether the cannabis-based treatment is effective [59,83].

### 3.6. Regulatory and Legal Issues with Cannabis

Several major government bodies, including the American Academy of Neurology and the American Academy of Pediatrics, do not recommend the use of cannabis in children with cancer due to limited evidence [59,108]. Currently in Canada, there are over 400 licensed providers of medical cannabis, and cannabis can be purchased from them in the form of dried cannabis, edibles, extracts, topicals, plants, and seeds [110].

## 4. Conclusions

Though a high number of good-quality studies exist that detail the anti-cancer effects of cannabis in adult tumors, the number for pediatric tumors is much lower. Given that various cannabinoids and even cannabis oils have been shown to be well tolerated in children for the treatment of CINV and epilepsy, there is good reason to pursue more preclinical and clinical research on the anti-cancer effects of cannabis in more pediatric tumors. This research needs to be done specifically for pediatric tumors, as their genetic and biological makeup often differs from that of their adult counterparts. This may also mean that the known mechanisms by which cannabis has anti-cancer effects in adult tumors may not apply exactly to pediatric tumors, and research is needed in this case to elucidate those mechanisms.

Existing data show that both the general public and physicians support this research. At the moment, a lack of policies around how and when cannabis can safely be used for pediatric patients is the main roadblock described by both parents and physicians when curious about the use of cannabis for pediatric patients. There now exist more than enough data on how cannabis is tolerated in children for such policies to be written. This will allow physicians and parents to make evidence-based decisions around cannabis use in children and will prevent parents from seeking advice from other, less reliable sources. This is especially relevant as cannabis is legalized in more and more places globally.

Legalization is another strong justification for more research into this area. It makes cannabis and potential misinformation around its use and effectiveness in pediatric populations more accessible to the general public. However, it may also be a positive, as legalization allows more strict regulation of and higher production standards for cannabis products. This may help prevent potential overdoses of cannabis and allows for more specific study of the effects of various cannabinoids, whether alone or in combination with each other or other components of cannabis, such as terpenes, alkenes, terpenoids, and flavonoids.

More widespread use of cannabis in pediatric patients will also allow a greater understanding of its developmental effects. While there is a good amount of data on the effects of cannabis on brain development in the AYA group and its effects on children exposed to cannabis in utero, there are few to no data about the effects of cannabis on children who receive it between the ages of 1 and 14. There are already studies investigating the late effects of current pediatric oncology treatments among pediatric cancer survivors, which could be used to study the late effects of any cannabis treatment as well. Furthermore, the severe and sometimes deadly immediate and late effects of existing pediatric cancer treatments necessitate the development of less toxic therapies.

Ideally, research on cannabis will reveal it as a highly effective pediatric cancer therapy without the severe side effects seen with currently accepted treatments. There is potential for cannabis to be used in a treatment regimen with currently existing treatments, such as surgery, chemotherapy, and radiotherapy. More research is needed to determine how it may interact with various drugs and other treatment modalities for it to be incorporated into pediatric cancer treatment. Additionally missing is an understanding of its anti-cancer action on a molecular level in pediatric cancer; this area necessitates its own study, as pediatric and adult cancers are often very different at the molecular level. This will help with predicting what tumors will respond to cannabinoid treatment and which combinations of cannabinoids will work best for each particular tumor type. Ultimately, research on the use of pediatric tumors is extremely well warranted and necessary, as it may genuinely represent a better option for children with improved survival and better outcomes for this patient population.

## Figures and Tables

**Table 1:** Summary of published clinical trials involving pediatric patients and some form of cannabinoid treatment.

Identifier	Title	Age of Patients (Years)	Disease	Study Type	Cannabinoid Drug Used	Concomitant Treatment?	Results	Side Effects	Ref
NCT02987114	A Phase II, Open-label, Single-center Clinical Study to Evaluate the Safety, Tolerability, and Efficacy of Oral Administration of PTL101	2–15	TRE	Interventional, single group assessment, open label	CBD capsules (PTL101), twice daily, 25 mg/kg/day up to 450 mg/kg/day	-	81.9% ± 24.6% reduction baseline median seizures, 73.4 ± 24.6% reduction monthly seizure frequency	Sleep disturbance/insomnia 25%, somnolence 18.8%, increased seizure frequency 18.8%, restlessness 18.8%, no SAE	[60]
NCT01898520	The Efficacy, Safety and Tolerability of Sativex as an Adjunctive Treatment to Existing Anti-spasticity Medications in Children Aged 8 to 18 Years With Spasticity Due to Cerebral Palsy or Traumatic Central Nervous System Injury Who Have Not Responded Adequately to Their Existing Anti-spasticity Medications: a Parallel Group Randomised, Double-blind, Placebo-controlled Study Followed by a 24-week Open Label	8–18	CP or traumatic CNS injury	Interventional, parallel assignment, double-blind, placebo-controlled	Sativex (THC 27 mg/mL: CBD 25 mg/mL), dose of 2.7 mg THC and 2.5 mg CBD, up to 12 doses per day	-	No difference in caregiver-reported spasticity	39% had treatment-related adverse events, only 23% in placebo-controlled group	[61]
NCT02324673	A Phase ½ Study to assess the pharmacokinetics and safety of Multiple Doses of Pharmaceutical Cannabidiol Oral Solution in Pediatric Patients with Treatment-Resistant Seizure Disorders	1–17	TRS	Interventional, randomized, parallel assignment, placebo controlled, double blind	CBD at 10, 20, or 40 mg/kg/day	-	CBD levels variable across patients, but overall well tolerated.	Somnolence 21.3%, anemia 18%, diarrhea 16.4%; 3 (1.6%) SAEs related to study drug: skin rash.	[62]
NCT02091206	A Double Blind, Placebo-controlled, Two-part Study to Investigate the Dose-ranging Safety and Pharmacokinetics, Followed by the Efficacy and Safety of Cannabidiol (GWP42003-P) in Children and Young Adults With Dravet Syndrome	4–10	DS	Interventional, randomized, parallel assignment, placebo controlled, double blind	CBD (Epidiolex) at either 5, 10, or 20 mg/kg/day	CBZ, LVA, SPL, TPM, VPA	When taking AED with CBD, CBD did not affect AED levels	Pyrexia, somnolence, decreased appetite, sedation, vomiting, ataxia, and abnormal behavior. Two discontinued for AEs: pyrexia and rash, and elevated transaminases	[63]
NCT02091375	A Double Blind, Placebo Controlled Two-part Study to Investigate the Dose-ranging Safety and Pharmacokinetics, Followed by the Efficacy and Safety of Cannabidiol (GWP42003-P) in Children and Young Adults With Dravet Syndrome	2–18	DS	Interventional, randomized, parallel assignment, placebo controlled, double blind	Epidiolex 20 mg/kg/day	CBZ, LVA, SPL, TPM, VPA	A total of 43% of patients had 50% reduction in convulsive seizure frequency, frequency of all seizures was significantly reduced, excepting nonconvulsive seizures	Diarrhea, vomiting, fatigue, pyrexia, somnolence, and abnormal liver-function tests, more withdrawals in CBD group; 93% of CBD group experienced AE vs. 75% placebo, SAE for 16.4% CBD patients and 5.1% placebo	[64]
NCT02224703	A Randomized, Double-blind, Placebo-controlled Study to Investigate the Efficacy and Safety of Cannabidiol (GWP42003-P) in Children and Young Adults With Dravet Syndrome	2–18	DS	Interventional, randomized, parallel assignment, placebo controlled, double blind	CBD (Epidiolex) at either 10 or 20 mg/kg/day	-	Reduction from baseline in convulsive seizures: 48.7% for 20 mg/kg/day, 45.7% for 10 mg/kg/day, 29.8% placebo. Reduction from baseline in total seizure frequency: 47.3% for 20 mg/kg/day, 56.4% for 10 mg/kg/day, 29.7% placebo	A total of 88% experienced adverse events (89.9% 20 mg/kg/day, 87.% 10 mg/kg/day, 89.2% placebo); decreased appetite, diarrhea, somnolence, pyrexia, fatigue; 22.7% SAEs; 51.7% AEs resolved by end of trial for CBD, 60.3% resolved for placebo	[65]
NCT02224560	A Randomized, Double-blind, Placebo-controlled Study to Investigate the Efficacy and Safety of Cannabidiol (GWP42003-P; CBD) as Adjunctive Treatment for Seizures Associated With Lennox-Gastaut Syndrome in Children and Adults.	2–55	LGS	Interventional, randomized, parallel assignment, placebo controlled, double blind	Epidiolex, either 10 or 20 mg/kg/day	CBZ, LVA, SPL, TPM, VPA	Patients receiving 20 mg/kg/day had 41.9% reduction in drop seizures and those receiving 10 mg/kg/day had 37.2% reduction, placebo had 17.2% reduction	AEs occurred for 94% of 20 mg group, 84% of 10 mg group, and 72% of placebo group; 89% of all AEs mild (somnolence, decreased appetite, pyrexia, vomiting); 26 had SAE in either CBD group, 7 of which were related to CBD	[66]
NCT02224690	A Randomized, Double-blind, Placebo-controlled Study to Investigate the Efficacy and Safety of Cannabidiol (GWP42003-P) as Adjunctive Treatment for Seizures Associated with Lennox-Gastaut Syndrome in Children and Adults	2–55	LGS	Interventional, randomized, parallel assignment, placebo controlled, double blind	Epidiolex, 20 mg/kg/day	CBZ, LVA, SPL, TPM, VPA	Median reduction in monthly drop seizure frequency from baseline 43.9% in CBD group, 21.8% in placebo.	AEs in 86% of patients in CBD group, 69% in placebo group, most mild/moderate (diarrhea, somnolence, pyrexia, decreased appetite, vomiting).	[67]
NCT02224573	An Open-Label Extension Study to Investigate the Safety of Cannabidiol (GWP42003-P; CBD) in Children and Young Adults with Inadequately Controlled Dravet or Lennox-Gastaut Syndrome	Over 2 years and completed NCT02224960, NCT02224560, NCT02224703, NCT02091206, or NCT02091375	DS, LGS	Interventional, single group assignment, open label	Epidiolex, concentration not specified	CBZ, LVA, SPL, TPM, VPA	Median reduction from baseline in drop seizure frequency from 48–60% over 48 weeks, median reduction in monthly seizure frequency 48–57% over 48 weeks.	A total of 92.1% had adverse events, 32.5% mild, and 43.4% moderate (diarrhea, somnolence, convulsion); 9.6% discontinued due to AEs.	[68]
NCT02544763	A Double-blind, Randomized, Placebo-controlled Study to Investigate the Efficacy and Safety of Cannabidiol (GWP42003-P, CBD) as Add-on Therapy in Patients with Tuberous Sclerosis Complex Who Experience Inadequately Controlled Seizures	1–65	TCS	Interventional, randomized, parallel assignment, placebo-controlled, double-blind	CBD solution, either 25 or 50 mg/kg/day	CBZ, LVA, VPA, VGB	Percent reduction from baseline in seizures 47.5% for 50 mg/kg/day CBD, 48.56% for 25 mg/kg/day CBD, 26.5% for placebo	Diarrhea and somnolence occurred more for CBD groups than placebo, and more in 50 mg/kg/day group than 25 mg/kg/day group. All patients in 50 mg/kg/day group experienced AEs. SAEs in 14% 50 mg/kg/day, 21% 25 mg/kg/day, 3% placebo	[69]
NCT02956226	Cannabinoids for Behavioral Problems in Autism Spectrum Disorder: A Double Blind, Randomized, Placebo-controlled Trial with Crossover	5–21	ASD	Interventional, randomized, parallel assignment, placebo-controlled, double-blind	Mix of pure CBD and THC in 20:1 ratio, or whole-plant extract enriched in CBD and THC to 20:1 ratio. Dose maximum 10 mg/kg/day	-	No change in behavioral problems generally, but improvement of disruptive behavior 49% on whole-plant extract vs. placebo.	No SAEs. Common adverse events were somnolence (28% plant extract, 23% pure cannabinoids, 8% placebo) and decreased appetite (25% plant extract, 21% pure cannabinoids, and 15% placebo)	[70]

Abbreviations: “AE” (adverse event); “AED” (anti-epileptic drug); “ASD” (autism spectrum disorder); “CBZ” (Clobazam); “CNS” (central nervous system); “CP” (cerebral palsy); “DS” (Dravet syndrome); “LGS” (Lennox–Gastaut syndrome); “LVA” (Leviraccetam); “SAE” (serious adverse event); “SPL” (Stiripentol); “TCS” (tuberous sclerosis complex); “TPM” (Topiramate); “TRE” (treatment-resistant epilepsy); “TRS” (treatment-resistant seizures); “VPA” (Valproate), “VGS” (Vigabatrin).

**Table 2:** Currently ongoing or unpublished clinical trials involving pediatric patients and some form of cannabinoid treatment.

Identifier	Title	Patients	Disease	Study Type	Cannabinoid Drug Used	Status
NCT03467113	A study to assess the safety and tolerability of ZX008 in children and young adults with DS or LGS currently taking CBD	Children (2–18)	Dravet or Lennox–Gastaut syndromes	Interventional, single group assignment, open label	ZX008, fenfluramine hydrochloride 0.2 or 0.8 mg/day, but patients must also be taking CBD	ANR
NCT03024827	Cannabidiol in children with refractory epileptic encephalopathy (CARE-E)	Children (1–10)	Epileptic encephalopathy	Interventional, single group assignment, open label	Cannimed 1:20 (CBD:THC)	ANR
NCT02523183	The use of medicinal cannabinoids as adjunctive treatment for medically refractory epilepsy	Children and young adults (0–20)	Medically refractory epilepsy	Observational, cohort, prospective	Medical cannabis	ANR
NCT02983695	Cannabinoid Therapy for Pediatric Epilepsy	Children (1–18)	Dravet syndrome	Interventional, single group assignment, open label	TIL-TC150 (contains THC and CBD)	ANR
NCT02660255	Safety and Tolerability of Cannabidiol in Subjects with Drug-Resistant Epilepsy	Children and adults (1–60)	Drug-resistant epilepsy	Expanded access	CBD (epidiolex)	AfM
NCT02397863	Epidiolex and Drug-Resistant Epilepsy in Children	Children (1–18)	Drug-resistant epilepsy (excluding Dravet Syndrome or Lennox–Gastaut Syndrome)	Expanded access	CBD (epidiolex, 25 mg/kg/day, up to 50 mg/kg/day)	A
NCT03196934	Expanded use of cannabidiol oral solution	Children (1–18)	Treatment-resistant seizure disorder	Expanded access	CBD	A
NCT03676049	Cannabidiol for drug-resistant pediatric epilepsy (expanded access use)	Children and young adults (5–19)	Drug-resistant epilepsy	Expanded access	CBD, between 2.5 and 7.5 mg/kg/day	A
NCT02987114	A study to evaluate the safety, tolerability, and efficacy of oral administration of PTL101 (Cannabidiol) as an adjunctive treatment for pediatric intractable epilepsy	Children (2–15)	Refractory epilepsy	Interventional, single group assignment, open label	Cannabidiol (PTL101), capsules taken twice daily. Capsules are either 50 or 100 mg and taken up to 25 mg/kg/day or up to 450 mg/kg/day	CNP
NCT02286986	Cannabidiol (CBD) to 27 patients (aged 2–19 years) with drug-resistant epilepsy	Children and young adults (2–25)	Drug-resistant epilepsy	Interventional, open label, single group assignment	Cannabidiol	CNP
NCT01898520	A safety, efficacy, and tolerability study of Sativex for the treatment of spasticity in children aged 8–18 years	Children (8–18)	Patients with CP or traumatic CNS injury with non-progressive spasticity	Interventional, parallel assignment, double-blind, placebo-controlled	THC:CBD 27:25 mg/mL spray (Sativex), given 100 uL per spray up to 12 per day	CNP
NCT02224573	GWPCARE5- an open-label extension study of cannabidiol (GWP42003-P) in children and young adults with Dravet or Lennox–Gastaut syndrome	Children and adults (2+)	Lennox–Gastaut syndrome, Dravet syndrome	Interventional, single group assignment, open label	Epidiolex	CNP
NCT02953548	Trial of cannabidiol (CBD; GWP42003-P) for infantile spasms	Children (0.083–2)	Infantile spasms and hypsarrhythmia	Interventional, single group assignment, open label	Epidiolex, 40 mg/kg/day	CNP
NCT02954887	Phase 3 trial of cannabidiol (CBD; GWP42003-P) for infantile spasms: open-label extension phase (GWPCARE7)	Children (0.083–2)	Infantile spasms and hypsarrhythmia	Interventional, single group assignment, open label	Epidiolex, 40 mg/kg/day	CNP
NCT02073474	An observational post-marketing safety registry of Sativex	All ages (including children)	Anyone taking Sativex	Observational, prospective	Sativex (27 mg/mL THC, 25 mg/mL CBD)	CNP
NCT02229032	Genetic analysis between Charlotte’s web responders versus non-responders in a Dravet population	Children and adults (0–50)	Dravet syndrome	Observational, cohort, cross-sectional	Charlotte’s web strain of medical marijuana	CNP
NCT04406948	Study of safety and efficacy of MGCND00EP1 as an add-on treatment in children and adolescents with resistant epilepsies	Children (1–18)	Drug-resistant epilepsy	Interventional, randomized, parallel assignment, placebo controlled, double blind	MGCND00EP1 (cannabis oil with 20:1 CBD:THC ratio), up to 25 mg/kg/day	NYR
NCT02332655	Cannabidiol expanded-access study in medically refractory Sturge–Weber syndrome	Children and adults (0.083–45)	Refractory Sturge–Weber syndrome	Interventional, single group assignment, open label	Epidiolex 2 mg/kg/day up to 25 mg/kg/day	NYR
NCT04485104	Safety, pharmacokinetics, and exploratory efficacy assessment of adjunctive cannabidiol oral solution (GWP42003-P) compared with standard-of-care antiepileptic therapy in patients 1 to <12 months of age with tuberous sclerosis complex who experience inadequately controlled seizures	Infants (1–11 mo)	Tuberous sclerosis complex	Interventional, randomized, parallel assignment, open label	Epidiolex	NYR
NCT02783092	A double-blind trial to evaluate efficacy and safety of cannabidiol as an add-on therapy for treatment in refractory epilepsy	Children (2–18)	Refractory epilepsy	Interventional, randomized, double-blind, placebo-controlled	CBD in corn oil, 5–25 mg/kg/day	R
NCT03848481	CBDV vs. placebo in children and adults up to age 30 with Prader–Willi syndrome	Children and young adults (0–30)	Prader–Willi syndrome	Interventional, randomized, parallel assignment, placebo controlled, double blind	CBDV (cannabidivarin) compound at 10 mg/kg/day, contains <0.2% THC	R
NCT04611438	Research on cognitive effects of cannabidiol on Dravet syndrome and Lennox–Gastaut syndrome	Children (2–18)	Lennox–Gastaut syndrome, Dravet syndrome	Interventional, single group assignment, open label	Cannabidiol 5 mg/kg/day up to 10 mg/kg/day	R
NCT03196466	Population pharmacokinetics of antiepileptic in pediatrics (EPIPOP)	Children (0–18)	Epilepsy	Observational, cohort, retrospective	CBD	R
NCT03052738	Medical marijuana in the pediatric central nervous system tumor population	Children (2–18)	Central nervous system tumor	Observational, cohort, prospective	Medical marijuana	R
NCT03848832	Efficacy and Safety of Cannabidiol Oral Solution (GWP42003-P, CBD-OS) in Patients with Rett Syndrome (ARCH)	Children (2–18)	Rett Syndrome	Interventional, randomized, parallel assignment, double blind	Epidiolex 100 mg/mL twice a day	ANR
NCT00153192	Study to Evaluate the Efficacy of Dronabinol (Marinol) as an Add-On Therapy for Patients on Opioids for Chronic Pain	Anyone (includes children)	Chronic pain average score 4/10 or more	Interventional, randomized, crossover assignment, double blind	N/A	CNP
NCT03614663	Clinical Study of caNNabidiol in childrEn and adolesCenTs With Fragile X (CONNECT-FX)	Children (3–17)	Fragile X Syndrome (FMR1 full mutation)	Interventional, randomized, parallel assignment, double blind	ZYN002—CBD clear transdermal gel; patients up to 35 kg get 125 mg CBD, above that get 250 mg CBD	CNP
NCT03824405	Study of the Safety, Tolerability, and Efficacy of BTX 1204 in Patients with Moderate Atopic Dermatitis	Children and adults (12–70)	Atopic Dermatitis	Interventional, randomized, parallel assignment, double blind	BTX 1204 (synthetic cannabinoid)	CNP
NCT03573518	Evaluation of BTX 1503 in Patients with Moderate to Severe Acne Vulgaris	Children and adults (12–40)	Acne Vulgaris	Interventional, randomized, parallel assignment, double blind	BTX 1503 (synthetic cannabinoid)	CNP
NCT03699527	Medical Cannabis Registry and Pharmacology (Med Can Autism)	Children and young adults (1–21)	Autism	Observational, cohort, prospective	Any cannabis product	CNP
NCT04252586	An Open-Label Extension Study of Cannabidiol Oral Solution (GWP42003-P, CBD-OS) in Patients with Rett Syndrome (ARCH)	Children (2–18)	Have completed RCT GWND18064 (NCT02848832)	Interventional, single group assignment, open label	Epidiolex 100 mg/mL twice a day	EBI
NCT03734731	Cannabis vs. Opioid Pain Management: Objective Testing Comparisons	Children and adults (0–90)	Pain	Interventional, single group assignment, open label	Any cannabis product	NYR
NCT04520685	CAnnabidiol Study in Children with Autism Spectrum DisordEr (CASCADE)	Children (5–17)	Autism Spectrum Disorder	Interventional, randomized, crossover assignment, double blind	CBD up to 10 mg/kg/day	NYR
NCT04721691	Efficacy of Epidiolex in Patients with Electrical Status Epilepticus of Sleep (ESES)	Children (2–17)	Electrical status epilepticus of sleep	Interventional, randomized, crossover assignment, double blind	Epidiolex up to 20 mg/kg/day	NYR
NCT04517799	Trial of Cannabidiol to Treat Severe Behavior Problems in Children With Autism	Male children (7–14)	Autism	Interventional, randomized, crossover assignment, double blind	Epidiolex up to 20 mg/kg/day	R
NCT03944447	Outcomes Mandate National Integration with Cannabis as Medicine for Prevention and Treatment of COVID-19 (OMNI-Can)	Children and adults (7+)	Clinical diagnosis of a qualifying condition for medical marijuana	Interventional, non-randomized, single group assignment, open label	Medical cannabis, must be legal in patient’s state	R
NCT03866941	Acute and Chronic Toxicity of Some Synthetic Cannabinoids in Assiut Psychiatric Hospitals	Anyone (includes children)	Admitted to psychiatric facility for cannabinoid toxicity	Observational, prospective	Any cannabis product	R
NCT03900923	Cannabidiol for ASD Open Trial	Children (7–17)	Autism Spectrum Disorder	Interventional, single group assignment, open label	CBD 3, 6, or 9 mg/kg/day	R
NCT03802799	Open-Label Extension to Assess the Long-Term Safety and Tolerability of ZYN002 in Children and Adolescents with FXS	Children (3–18)	Have completed NCT03614663	Interventional, single group assignment, open label	ZYN002—CBD clear transdermal gel	R
NCT04447846	Novel Cognitive Treatment Targets for Epidiolex in Sturge–Weber Syndrome	Children and adults (3–50)	Sturge–Weber syndrome	Interventional, single group assignment, open label	Epidiolex up to 20 mg/kg/day	R
NCT04634136	Full-Spectrum Medical Cannabis for Treatment of Spasticity in Patients with Severe Forms of Cerebral Palsy (HemPhar)	Children and young adults (5–25)	Cerebral palsy with spasticity	Interventional, randomized, crossover assignment, double blind	HemPhar cannabis extract THC:CBD 1:10	R

Abbreviations: “ANR” (active, not recruiting); “AfM” (approved for marketing); “A” (available); “CNP” (completed, not published); “NYR” (not yet recruiting); “R” (recruiting); “EBI” (enrolling by invitation).

**Table 3:** Summary of available preclinical evidence of anti-cancer activity in pediatric cancer models.

Cancer Type	Cannabinoid Used/ECS Component Investigated	Experiment Details	Proposed Mechanism	Ref
Pediatric low-grade glioma	CB1 receptor levels	Tumor samples from 33 patients	High *CNR1* levels seen at diagnosis in tumors that remained stable or underwent spontaneous involution, implying that CB1 receptor activation may participate in this phenomenon.	[90]
Brain tumors	CB2 receptor levels	Tumor samples from 20 adult patients and 25 pediatric patients	CB2 levels are higher in more malignant tumors and in areas of tumors with very proliferative/invasive cells.	[91]
Neuroblastoma	CBD	SK-N-SH	CBD reduces proliferation, induces apoptosis, and increases caspase-3 levels of SK-N-SH cells in vitro and in vivo.	[92]
Neuroblastoma	CBD	SH-SY5Y IMR32	CBD altered expression of many miRNAs, including upregulation of has-let-7a and downregulation of has-miRNA-1972. These were confirmed to be responsible for increases in casp-3 and GAS-7 (by has-let-7a) and decreases in BCL2L1, SIRT1, and MYCN (by has-miRNA-1972). CBD also decreased AKT1 and increased PTEN, caused apoptosis, inhibited cell migration, and shifted the cellular metabolism towards glycolysis.	[93]
Neuroblastoma	AM404	SK-N-SH	AM404 inhibits the transcriptional activity of both NFAT and NF-κB through CB1 and TRPV1-independent pathways. AM404 also inhibits invasion and decreases expression of MMP-1, 3, and 7.	[94]
Leukemia	CBD	Jurkat, EL4 (mouse lymphoma), MOLT-4 (adult ALL)	CBD decreases cell viability and increases apoptosis, both in vitro and in vivo. In vitro only, CBD increased PARP and caspase-3 cleavage, decreased mitochondrial membrane potential, and increased ROS, all of which were dependent on CB2 receptor activation. The CBD-induced decrease in p-p38 MAPK also depended on ROS production.	[95]
Leukemia	THC	Jurkat cells	THC triggers extrinsic (cleavage of casp 8, 10) and intrinsic (mitochondrial membrane potential, cytochrome c release, cleavage of casp 3, 9) apoptotic pathways, but primarily the intrinsic pathway is responsible.	[96]
Leukemia	THC	Jurkat cells	THC downregulated Raf-1/MEK/ERK signaling pathway by altering phosphorylation status, causing BAD to relocate to mitochondria and triggering intrinsic apoptosis.	[97]
Leukemia	THC	Jurkat cells	THC induced apoptosis via CB2 receptor and increase in de novo ceramide production upstream of the intrinsic apoptotic pathway (mitochondrial hyperpolarization and cytochrome c release).	[98]
Leukemia	CPP95540	Jurkat cells Patient samples (T-ALL)	Apoptosis induced via production of ROS and activation of the mitochondrial intrinsic apoptosis pathway. This is not mediated via CB1 or CB2 receptors. This occurred both in an established cell line and in bone marrow samples from three T-ALL patients.	[99]
Leukemia	CBD	Pediatric Leukemic lines: Jurkat (T-ALL), CCFR-CEM (T-ALL) Adult Leukemic lines: MOLT-3 (T-ALL), K562 (CML), Reh (B-ALL), and RS4;11 (B-ALL)	High concentrations of CBD inhibit migration and increases apoptosis and autophagy at high concentrations in T-ALL cells while low CBD concentrations increase proliferation. CBD directly increases mitochondrial Ca^2+^, which induces cytosolic 2+ and leads to apoptosis. CBD also increases ROS levels and activates caspases 3 and 9.	[100]
Chronic lymphocytic leukemia	AM251 ACEA JWH133 AM630 R-(+)-methanandamide CBD	Patient-derived cell cultures	High CNR1 expression was correlated with poorer outcomes. Treatment with CB1 and/or CB2 agonists and antagonists did not alter viability or invasion significantly in patient cells or normal peripheral blood lymphocytes	[55]
Leukemia	CBD, CBG, THC	CEM (ALL, pediatric) HL60 (promyelocytic leukemia, adult)	Combinations of any 2/3 cannabinoids are better at reducing cell viability than any cannabinoid alone, particularly combinations with CBD, especially the CBD/THC combination. These combinations worked synergistically with vincristine and cytarabine, but the synergy was better when cells were treated with cytarabine or vincristine before cannabinoid treatment.	[43]
Rhabdomyosarcoma	THC, HU-210, Met-F-AEA	Rh4, Rh28 (translocation positive rhabdomyosarcoma) RMS13, RD, MRC-5 (lung fibroblasts)	Cannabinoid treatment decreased viability of translocation-positive rhabdomyosarcoma (tposRMS) mediated through the CB1 receptor. This was via the Akt/ERK pathway and required the upregulation of p8.	[101]
